# Latch On: A protocol for a multi-centre, randomised controlled trial of perinatal support to improve breastfeeding outcomes in women with a raised BMI

**DOI:** 10.1016/j.conctc.2021.100767

**Published:** 2021-04-08

**Authors:** Sharleen L. O'Reilly, Eileen C. O'Brien, Denise McGuinness, John Mehegan, Barbara Coughlan, Denise O'Brien, Marcelina Szafranska, Sophie Callanan, Shenda Hughes, Marie C. Conway, Mary Brosnan, Lucille Sheehy, Rosie Murtagh, Lorraine O'Hagan, Stephanie Murray, Charmaine Scallon, Elizabeth Dunn, Paula Power, Marie Woodcock, Amy Carroll, Marie Corbett, Michelle Walsh, Regina Keogh, Fionnuala M. McAuliffe

**Affiliations:** a UCD Perinatal Research Centre, School of Medicine, University College Dublin, National Maternity Hospital, Dublin, Ireland; b University College Dublin, Dublin, Ireland; c National Maternity Hospital, Dublin, Ireland; d Wexford General Hospital, Co Wexford, Ireland; e St.Luke's General Hospital, Co Kilkenny, Ireland; f Midlands Regional Hospital Mullingar, Co Westmeath, Ireland

**Keywords:** Breastfeeding, Overweight, Obesity, Randomised controlled trial, BMI, Body mass index, DUB, Dublin, WEX, Wexford, KIL, Kilkenny, MUL, Mullingar, BAPT, Breastfeeding Attrition Prediction Tool, IBCLC, International Board Certified Lactation Consultant, CHO, Community health organisations

## Abstract

**Introduction:**

Breastfeeding is associated with improved maternal and child outcomes. Women with a higher body mass index (BMI), who comprise about 50% of the population, are at increased risk of poorer breastfeeding practices and are a population who would benefit from breastfeeding.

**Methods:**

This protocol is for a multi-centre, randomised controlled trial of perinatal breastfeeding support among primiparous women with a BMI >25 kg/m^2^, using a previously-tested, multi-component intervention. The primary outcome is any breastfeeding at 3 months. The intervention will support mothers and their partners and spans from late pregnancy to six weeks postpartum. Intervention components include group antenatal breastfeeding education, individual face-to-face education in the immediate postnatal period, professional support to six weeks’ postpartum and weekly phone calls in the immediate postpartum period from an International Board Certified Lactation Consultant (IBCLC). The intervention will target attitudes towards breastfeeding, breastfeeding self-efficacy, and subjective norms around infant feeding with the aim to normalise the behaviour.

**Results:**

We anticipate that the intervention will be well-accepted and feasible to carry out within four maternity units in the East of Ireland. Furthermore, essential formative qualitative work has been conducted to inform the intervention design and to ensure that it is contextually appropriate.

**Conclusion:**

The proposed intervention will be invaluable to policy-makers in providing insights into what specific interventions are effective in improving breastfeeding rates for women with a raised BMI.

## 1 Introduction

Breastfeeding provides optimal nutrition for infants and children from birth to two years and beyond [1]. For a woman, the benefits include supporting her in returning to her pre-pregnancy body mass index (BMI), better pregnancy spacing, and a lower risk of breast cancer and diabetes later in life [2]. The infant benefits include lower long-term risk of developing obesity and type 2 diabetes [2]. Wider community benefits of increased breastfeeding are gained through increased parent presenteeism at work and longer term better health in the woman [2]. Irish breastfeeding rates are amongst the lowest worldwide [2,3] with only 60% of Irish newborns receiving any breastmilk and of that percentage, 50% are exclusively breastfed on discharge from the maternity services [4]. Other developed countries such as Australia, have 96% breastfeeding on discharge and exclusivity rates of 62% at four months [5]. Health promotion strategies are in place to support increased breastfeeding and the Irish National Breastfeeding Action plan documents the need for a 2% year-on-year increase in any breastfeeding if any progress is to occur [6].

Women with overweight or obesity are an identified population sub-group that have low breastfeeding success [7]. While breastfeeding initiation rates in women with a raised BMI are 13% lower than women with a healthy BMI [7], the drop off is steep once breastfeeding has started. Women with overweight or obesity are 76% and 83% less likely to be BF at 6 weeks and 6 months, respectively, compared to women with a healthy BMI [8]. Successful multifaceted breastfeeding support intervention elements include: health professional-led education, non-health professional-led counselling and peer support interventions [9]. Interventions embedded into standard care; ongoing scheduled postpartum visits; tailored interventions to population needs; interventions delivered by health professional or peers or a mix of both; and face-to-face support improved longer term breastfeeding outcomes [10]. A recent pilot of a multidimensional breastfeeding intervention was successfully carried out among 100 women, representing all weight classifications, at The National Maternity Hospital, Dublin and Wexford General Hospital, Wexford [11,12]. Primiparous women received an antenatal group education class, a one-to-one International Board Certified Lactation Consultant (IBCLC) consultation after birth and access to online resources, follow-up phone calls from an IBCLC, and a breastfeeding support group. The intervention found exclusive breastfeeding at 3 months was increased compared to national prevalence, well-accepted and feasible to carry out in both urban and rural areas [11]. The pilot identified that mothers with a BMI >25 kg/m^2^ were less likely to exclusively breastfeed at 3 months [12] and were a potentially important sub-group to target within a fully powered randomised trial.

This study aims to investigate the effectiveness of a multicomponent breastfeeding support intervention on the prevalence of any breastfeeding at 3 months among women with a BMI >25 kg/m^2^. It is hypothesised that women in the intervention group will have higher prevalence of breastfeeding initiation, longer breastfeeding duration and higher breastfeeding self-efficacy than those in the control group. It is also anticipated that the inclusion of the support partner will improve the support-structure for women in their home setting.

## 2 Methods

### 2.1 Trial design

**Fig. 1:**
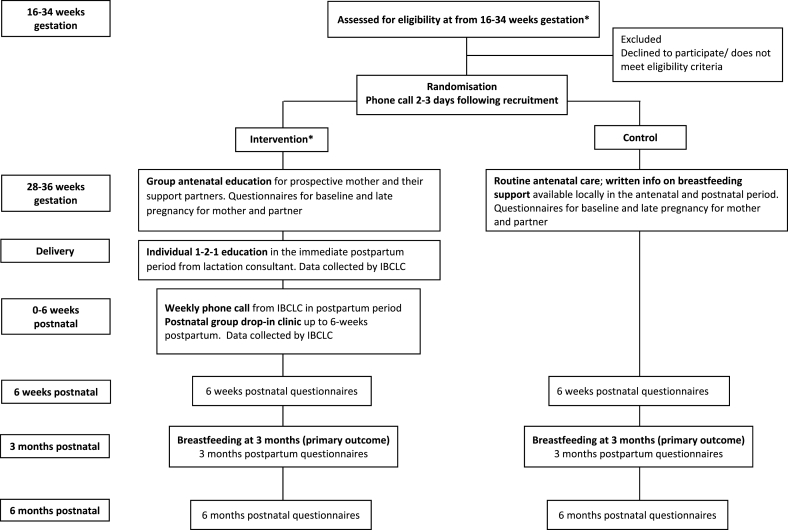
**Flow chart for The Latch On Study;** *recruitment and intervention impacted by covid-19 pandemic: March 12^th^, 2020 until present (April 2020, and likely this will be the case until recruitment is completed); face-to-face recruitment did not continue during this period, face-to-face antenatal classes were also suspended during this time with online classes offered as an alternative. Drop-in clinics were available by appointment only.

Latch On is a multicentre randomised clinical trial to test the effectiveness of an education and support intervention program, for women with overweight or obesity, during pregnancy and the first six weeks postnatal on increasing breastfeeding prevalence at three months postpartum. The flow of participants through the study from recruitment to completion is illustrated in Fig. 1. Ethical approval was granted by National Maternity Hospital Ethics Committee in December 2018 (ref: EC.24.2018). The trial was prospectively registered (ISRCTN14819650), initial planning started on August 1^st^, 2018 and recruitment commenced on June 4^th^, 2019. Recruitment from March 12^th^, 2020 to date (April 2021) has been impacted by the covid-19 pandemic.

### 2.2 Recruitment and eligibility

#### 2.2.1 Recruitment

Recruitment began on June 4^th^, 2019 and the anticipated end date is November 2022. The recruitment sites are: National Maternity Hospital, Dublin (NMH); Wexford General Hospital, Wexford (WGH); St Luke's General Hospital, Kilkenny (SLK); and Midlands Regional Hospital, Mullingar (MRH). The project is part funded by the HSE Nursing and Midwifery Planning and Development Unit Dublin South, Kildare and Wicklow and sponsored by 10.13039/501100001631University College Dublin (UCD).

The NMH, based in the city centre of Dublin, Ireland's capital, is one of the largest maternity hospitals in Europe with approx. 9000 births per year. WGH is a smaller regional hospital in Wexford, Ireland, with approximately 2000 babies delivered each year. Similarly, SLK and MRH are regional hospitals with approximately 1600 and 1900 births each year respectively.

Recruitment strategies vary due to site-level differences in staffing, care delivery and timing of routine visits and include recruitment within antenatal clinics, with researchers or midwives approaching women in early pregnancy to the beginning of the third trimester. Posters are displayed throughout hospital maternity units and on hospital websites. Social media, including Twitter and Instagram are also used as a recruitment channel. At all sites, lists of the reasons for refusal to take part in the study are documented to keep a record of why participants did not wish to take part. Women were also free to decline participating without providing an explanation.

The COVID-19 pandemic emerged during the study and recruitment was adapted. Participants recruited between June 4^th^, 2019 and March 12^th^, 2020 were enrolled prior to covid-19 (n = 105); all participants recruited after 12th March 2020 were enrolled during the covid-19 pandemic. Staff were limited in the time they could allocate to assist with the study during the pandemic, and therefore recruitment was mainly via self-referral from in-house notices and hospital websites, with pregnant women interested in the study contacting the research team about participating. Recruitment strategies were adapted with face-to-face recruitment in antenatal clinics reduced. At the NMH, the study was publicised in antenatal clinics via posters, through social media such as Twitter and Instagram, and also promoted at online antenatal classes. The charts of women attending antenatal clinics are now screened by researchers for eligibility, and those deemed eligible subsequently contacted via phone and provided with verbal information on the study. If they are interested in taking part, further information and consent forms are sent to them via email. At the regional hospital sites, the study was also publicised via posters, through social media, and promoted at online antenatal classes. Recruitment phone calls were also adapted at these sites where possible.

Face-to-face antenatal classes were moved to online virtual classes with follow-up phone calls offered as an alternative. Postnatal 1-1 consults were possible with IBCLC's at each hospital site. Postnatal breastfeeding drop-in clinics were also cancelled, and these clinics were instead offered by appointment only.

An outline of the PICO (population, intervention, comparison, outcomes) for the Latch On Study is included in Table 1.

**Table 1:** PICO (population, intervention, comparison, outcomes) for the Latch On Study.

Population	Inclusion criteria • Primiparous women • Singleton pregnancy • BMI ≥25 kg/m^2^ at booking visit • Age ≥18 years • Good understanding of English • Ability to give informed consent • Have a support partner available and willing to participate if in intervention group Exclusion criteria • Preterm (<37 weeks' gestation) delivery • Any condition requiring medication that is contraindicated for breastfeeding
Intervention	Intervention components include: • Group antenatal education for prospective mothers and their support partners • Individual education for new mothers in the immediate postnatal period^a^ • Professional support for new mothers to six weeks postpartum
Comparison	Control group will receive: • Routine antenatal care • Oral and written information on antenatal and postnatal breastfeeding support available in the study site hospital and community
Outcome	Primary outcome: • Breastfeeding at 3 months postpartum Secondary outcomes: • Intention to breastfeed following the antenatal component of the intervention • Initiation rates of breastfeeding • Exclusive and any breastfeeding prevalence at hospital discharge, 6 weeks, 3 months, and 6 months postpartum • Maternal and support partner attitudes toward breastfeeding • Breastfeeding self-efficacy • A comparison of breastfeeding prevalence, duration, self-efficacy and support structures among women who delivered pre-pandemic and during the pandemic

a Immediate postnatal period: women were usually seen by an IBCLC anytime during their hospital stay.

#### 2.2.2 Assessment of eligibility

Depending on the setting, electronic health records or traditional paper charts are used to screen women for eligibility in advance of women attending their antenatal appointment. Information on general sociodemographics, gestational age, reproductive history and pre- and early gestational weight gain will be obtained through interview with the participant.

#### 2.2.3 Inclusion criteria

Women are eligible if they are aged 18 or older, are identified as having a BMI of 25 kg/m^2^ or higher at their first antenatal visit; primipara; singleton pregnancy; 26–34 weeks pregnant at recruitment; proficient in English; able to give informed consent; have an identifiable support partner available, and are willing to participate in the trial. Support partners could be the baby's father, the pregnant woman's male or female partner, the pregnant woman's mother or sister or a trusted friend.

#### 2.2.4 Exclusion criteria

Preterm delivery (<37 weeks’ gestation) and any known/identified breastfeeding contraindications, inclusive of any conditions requiring medication contraindicated for breastfeeding.

#### 2.2.5 Informed consent

When women are approached, either in person or via phone calls, the study protocol is explained to them and they are asked if they are interested in taking part. Participating women are asked to provide signed informed consent within the same clinic visit if possible when recruitment is in person. If they would like more time to consider the study, they are given time to review study information and a consent form to complete and return to researchers. Researchers schedule a follow-up phone call for 2–3 days after this.

#### 2.2.6 Randomisation

Women are allocated to either intervention or control using a computer-generated scheme. Randomisation is a 1:1 allocation and stratified by study site and BMI category (CSTAR, UCD). Given the nature of the intervention, neither the participants nor the researchers are blinded to allocation. Sealed opaque envelopes will be sequentially numbered and treatment will be allocated automatically through selection of the next available envelope. Researchers select a sealed envelope in numerical order, open the envelope and will then advise the woman of the group they have been allocated to. Women receive a phone call after they have provided consent to take part in the study.

#### 2.2.7 Control

Women assigned to the control group are provided with oral and written information on antenatal and postnatal support for breastfeeding that is available in the study site hospital and community, and will receive routine antenatal care. A summary of the information available to women in the control group is available in Appendix A.

#### 2.2.8 Intervention

This multi-component intervention targets prospective mothers and their support partners and spans the perinatal period from late pregnancy to six weeks’ postpartum. Intervention components include group antenatal education for prospective mothers and their support partners, individual education for new mothers in the immediate postnatal period, and professional support for new mothers to six weeks postpartum (Table 2). Women allocated to the intervention group attend a group antenatal education session usually between 28–36 weeks gestation with their support partner.

**Table 2:** Intervention approaches used.

Approach	Description	Timing
SMS texts	Welcome to the study text with links to online questionnaires. These texts are standardised to ensure all participants received the same text. SMS texts are sent to participants in both the intervention and control groups.	Post randomisation
Email	Welcome to the study email with links to online questionnaires. Also used to send links to late pregnancy and postnatal questionnaires (6 weeks, 3 months and 6 months postpartum). These emails are standardised with all participants receiving the same email. Emails are sent to participants in both the intervention and control groups.	Throughout the study
Phone call from researcher	Researchers will phone participants to randomise them to a study group, and also to schedule the antenatal class and to give reminders about completing the online questionnaires. Phone calls from researcher to participants occur for both the intervention and control groups.	Throughout the study
Group education	Antenatal class – the antenatal class covers a range of topics in relation to breastfeeding rates, the benefits of breastfeeding, the basics of breastfeeding, and breastfeeding support. The antenatal classes are delivered by lactation consultants. Pregnant women and their support partners are required to attend the antenatal class. Intervention group only.	28–36 weeks
Individual consultation	IBCLC will provide face to face advice and support after the birth of the infant. Intervention group only.	1–3 days postpartum
Phone call from IBCLC	IBCLC will phone mother weekly to discuss her progress and issues she is having. Self-efficacy prompts. Intervention group only.	Once per week for 6 weeks postpartum
Breastfeeding support group clinic	IBCLC led drop-in weekly clinic made available to mothers for the first 6 weeks postpartum. Intervention group only. Breastfeeding support clinic is by appointment only during the covid-19 pandemic.	Once per week for 6 weeks postpartum

During the covid-19 pandemic (from 12th March 2020 onwards), changes to the provision of the intervention components were made. All antenatal classes now take place virtually, and postnatal drop-in clinics are now taking place by appointment only. Women receive postnatal follow-up support over the phone from lactation consultants for the first 6 weeks postpartum, with an in-person appointment arranged if needed or requested.

The intervention has been mapped onto the COM-B behaviour change framework with a focus on attitudes to breastfeeding, changing subjective norms and perceived behavioural control (Appendix B). Attitudes toward breastfeeding are targeted by providing education in the antenatal and postnatal period to mothers and their support partners. Subjective norms around infant feeding are targeted by providing education to the pregnant woman's support partner, and by facilitating breastfeeding support groups postpartum which will be attended by other breastfeeding mothers, normalising the behaviour. The mother's perceived behavioural control of her ability to feed her baby (breastfeeding self-efficacy) is targeted by providing antenatal and postnatal education that is realistic and describes what to expect when breastfeeding, including potential challenges that may arise and how to manage these challenges. This education equips women with the skills and confidence to manage breastfeeding, increasing their self-efficacy.

#### 2.2.9 Antenatal class

Pregnant women and their support partners are asked to attend an antenatal class at 28–36 weeks gestation. This class covers a range of topics in relation to breastfeeding rates, the benefits of breastfeeding, the basics of breastfeeding, and breastfeeding support. The antenatal classes are delivered by lactation consultants. Attendance at the antenatal class is compulsory for the support partner and they will be provided with specific information on how they can support the breastfeeding mother. All support partners were encouraged to attend the class; however, if a support partner was unable to attend, this was recorded and they were provided with information to read through in their own time, or a second antenatal class was arranged for a time the partner could attend. A copy of the antenatal class checklist is included in Appendix C, which outlines the content covered at the class.

#### 2.2.10 Individual face-to-face education

After the birth of the infant, an IBCLC provides the participant with face-to-face advice and support. The IBCLC covers aspects such as breast assessment, infant oral assessment, what to expect in the first few days, how to attach and recognise the baby is effectively feeding, as well as other elements such as ensuring a minimum of 8 feeds per 24 h, and knowing how to hand express. Details of newborn feeding method are collected at this face-to-face session and a breastfeeding assessment is completed by the IBCLC. Any other issues or challenges are also assessed at this session. Finally, a LATCH assessment is completed by the IBCLC. The postnatal assessment checklist (Appendix D) gives details of what is covered at this education session. The preference is for an in-person consultation with an IBCLC. If a mother is discharged prior to a lactation review they receive a telephone consult as soon as possible after discharge. Video calls are also arranged if required. A note of how 1-2-1 consultations were delivered to participants is recorded.

### 2.3 Professional support up to six weeks postpartum

#### 2.3.1 Drop-in clinics

Before the covid-19 pandemic, drop-in clinics were offered in a group setting to all participants allocated to the intervention group at their maternity unit. The age of the baby, relevant medical and breastfeeding history and information on any breastfeeding challenges were discussed at this drop-in clinic. Self-efficacy and a latch assessment are also completed and notes of a plan and follow-up are recorded where relevant. Drop-in clinics are by appointment only during the covid-19 pandemic, or a video call arranged if they cannot attend in person. A drop-in clinic checklist can be found in Appendix E.

#### 2.3.2 Weekly phone calls

Education is reinforced by weekly phone calls in the postpartum period from an IBCLC to answer any questions. The IBCLC discusses the woman's progress and any issues she may be having. The lactation consultant records details in relation to breastfeeding history, and also notes any breastfeeding challenges that were discussed with the participant. Self-efficacy of the mother is also prompted, and notes for a plan and follow-up recorded where relevant. A follow-up phone call checklist is included in Appendix F.

### 2.4 Data collection

#### 2.4.1 Process and impact evaluation

Midwives and IBCLC are provided with individual logins for the Latch-On online platform. This allows them to schedule antenatal classes and drop-in clinics in conjunction with the researchers at the primary study site (UCD/NMH). Process checklists to be completed by IBCLC include the antenatal class checklist, and the drop-in clinic checklist. Pregnancy outcome and pregnancy complications will be collected from hospital charts as per our usual pregnancy studies.

### 2.5 Outcome measures

#### 2.5.1 Primary outcome

The primary outcome is feeding method at 3 months' postpartum, assessed using self-reported questionnaires completed by the mother, including the Questionnaire on Infant Feeding by O'Sullivan et al. [13].

#### 2.5.2 Secondary outcomes

The secondary outcomes include:

1. Intention to breastfeed following the antenatal component of the intervention, assessed by open ended and closed ended questions in a questionnaire completed by the mother at pre-antenatal class
2. Initiation rates of breastfeeding, collected using hospital discharge data at discharge from hospital
3. Exclusive and any breastfeeding prevalence assessed using a questionnaire completed by the mother (including Questionnaire on Infant Feeding (O'Sullivan et al., 2017) [13]), at hospital discharge, 6 weeks', 3 months' and 6 months' postpartum.
4. Maternal and support partner attitudes toward breastfeeding, assessed by open ended and closed ended questions in a questionnaire completed by the mother at pre-antenatal class, 6 weeks', 3 months', and 6 months' postpartum.
5. Breastfeeding self-efficacy, assessed using Breastfeeding Self-Efficacy Short Form (Dennis, 2003) [14], at pre-antenatal class, 6 weeks', 3 months', and 6 months' postpartum
6. Additional outcome related to COVID-19 pandemic: A comparison of breastfeeding prevalence, duration, self-efficacy and support structures among women who delivered pre-pandemic (June 2019–Feb 2020) and during the pandemic (March 2020-TBC).

### 2.6 Measures of intervention exposure

#### 2.6.1 Baseline and follow-up measurements [outcome]

Women and their partners complete the below antenatal questionnaires at baseline and late pregnancy. Where possible, validated questionnaires are used. If a validated questionnaire was not available, a self-designed questionnaire was used. The women's questionnaires include measurement of:

• Breastfeeding (Attitude to breastfeeding)
• Feeding Confidence
• Infant Feeding Attitude
• Breastfeeding Attrition Prediction Tool (BAPT) Survey
• Breastfeeding Support

The support partner completes the below antenatal questionnaires at baseline and late pregnancy:

• Breastfeeding (Attitude to breastfeeding)
• Breastfeeding Support

Women and their partners are sent an email with a link to their questionnaires, and the researchers make a follow-up call if required to remind them to complete these.

Mothers complete a range of questionnaires at 6 weeks, 3 months, and 6 months postpartum to collect information on Feeding Details, Feeding Decision, Breastfeeding Experiences, Feeding Confidence, Infant Feeding Attitude, Breastfeeding Support, Cost Diary, Quality of Life, and Postnatal Depression.

### 2.7 Intervention evaluation [process] from the participant and lactation consultant perspectives

Exit interviews will be conducted with both women and their partners (where available) to explore barriers and enablers to engaging in the intervention. The interviews will be semi-structured and the COM-B framework will be applied to the data analysis.

The normalization of the intervention into routine practice will be explored using the NOMAD questionnaire pre- and post-intervention [15]. A focus group will be conducted at the completion of the study to explore IBCLC perspectives on the sustainability of the intervention and contextual factors involved in delivery.

### 2.8 Sample size

In 2017, prevalence of any breastfeeding at 3 months was 38.6% in Community Health Organisation 5 (CHO5) (SLK and WGH are within this CHO); 32.4% in CHO8 (MRH is within this CHO) and 37.3–45.7% in CHO 6, 7 and 9 (catchment area for NMH). Following our pilot study, 60–70% of women had any breastfeeding at 3 months [11]. Given that the women in this study will all have a BMI >25 (less likely to breastfeed), but will volunteer for a breastfeeding research study (more likely to breastfeed), we anticipate that the baseline breastfeeding rate within the control group would be approximately 40% at 3 months’ postpartum. This estimate is derived from a pilot study conducted in The National Maternity Hospital, Dublin from February to July 2016, and in Wexford General Hospital, Wexford from July to November 2016. We anticipate that the intervention will increase breastfeeding at 3 months to 60–70%. We based our sample size calculation on an uncorrected Pearson chi-square test of the primary hypothesis at the 0.05 level with 80% power using the PS Power and Sample Size package (CSTAR, UCD). To observe an increase in prevalence from 40 to 60%; we estimate that 194 women would be required to have 80% power to detect this effect size at a significance of 0.05, that is, 97 in each arm. To observe an increase in prevalence from 50 to 70%; we estimate that 186 women would be required to have 80% power to detect this effect size at a significance of 0.05, that is, 93 in each arm. To allow for drop outs, and allowing for the block randomisation procedure stratified by site, we calculated the required sample size will involve recruiting 220 women across the four hospital sites.

### 2.9 Statistical analysis

The primary analysis of the trial will comprise of a comparison of the primary endpoint using a logistic regression model, stratified by site. A conclusion of efficacy will be determined by a statistically significant effect at a 0.05 level on the intervention coefficient. The predicted absolute probabilities of any breastfeeding, and an odds ratio for the intervention effect will be presented, overall and by site, with 95% confidence intervals. As a sensitivity analysis, a random effects logistic model will be fitted with a random intercept per site to allow for a distribution of baseline rates; however, we expect the small number of sites will be inadequate for stable or precise variance component estimates of this parameter, and it remains a secondary sensitivity analysis.

## 3 Discussion

This is one of the few randomised controlled trials designed to improve breastfeeding outcomes in women with obesity and overweight by intervening both in the antenatal and postnatal periods. The multifaceted approach has been mapped onto the COM-B behaviour change framework [16] and reflects best practice in complex implementation study design [17]. The covid-19 pandemic resulted in challenges to recruitment and provision of the study; however, adaptations were made to the protocol to allow the study to proceed.

## Funding

The project is part funded by the HSE Nursing and Midwifery Planning and Development Unit Dublin South, Kildare and Wicklow and sponsored by 10.13039/501100001631University College Dublin.

## Declaration of competing interest

The authors declare that they have no conflict of interests.
