# Supplementary material for: Latch On: A protocol for a multi-centre, randomised controlled trial of perinatal support to improve breastfeeding outcomes in women with a raised BMI
Source: Contemp Clin Trials Commun. 2021 Apr 8;22:100767. doi: 10.1016/j.conctc.2021.100767 (PMC8165542; doi:10.1016/j.conctc.2021.100767)
Supplement: Multimedia component 1 [file mmc1.docx]

**Appendix A**

**Information provided to the control group**

Support offered to the control group is summarised below:

- Women in the control group can attend a breastfeeding antennal class that is offered to all pregnant women as part of routine antenatal care
- Further information is available for women in the control group including leaflets, online information, and video clips relating to breastfeeding
- Information leaflets/booklets covered topics such as feeding cues, feeding in the first few days, attaching baby to the breast and hand expressing
- Women are also free to avail of any additional supports they planned to themselves

**Appendix B: COM-B mapping of The Latch On Study**

| **COM-B** | **Intervention functions** | **Intervention women need to…** | **Intervention partners need to…** |
| --- | --- | --- | --- |
| Capability | |  |  |
| Knowledge | Educate about ways of enacting the desired behaviour or avoiding the undesired one | Know why breastfeeding is important and how to do it | Know why breastfeeding is important and how it is done |
| Skill | Train in cognitive, physical or social skills | Learn how to breastfeeding and negotiate everyday problems such as maintaining social support | Learn how to support their partner to have opportunity, capability and motivation to breastfeeding in the home and social settings |
| Strength | Train or Enable development of mental or physical strength required for the desired behaviour or to resist the undesired one | Build self-efficacy and physical health for breastfeeding | Build self-efficacy on supporting partner to breastfeeding |
| Stamina/Endurance | Train or Enable endurance required for the desired behaviour or sustained resistance undesired one | Build self-efficacy for longer term breastfeeding over time in the face of a formula fed-culture | Build self-efficacy for longer term breastfeeding over time in the face of a formula fed-culture |
| Opportunity | |  |  |
| Time | Train or Restructure the environment to reduce time demand or competing time demands for desired behaviour (and additionally use Restriction to reduce undesired behaviour) | Create dedicated time for breastfeeding (partner support essential) | Create dedicated time for breastfeeding support |
| Resources | Restructure the environment to increase social support and cultural norms for desired behaviour (and additionally use Restriction to reduce undesired behaviour) | Acquire suitable breastfeeding supports if needed e.g. pillow, pump, creams, shields, clothing | Support the restructure of environments e.g. home, for breastfeeding |
| Location/Physical barriers | Train or Restructure the environment to provide cues and prompts for desired behaviour (and conversely for undesired behaviour) | Identify ways to make breastfeeding easier so can access facilities when needed e.g. at work, out socially or reminders at strategic times | Support removal of barriers to breastfeeding in settings |
| Interpersonal influences/cultural expectations | Restructure the social environment or use Modelling to shape people’s way of thinking | Have supporters around them | Model breastfeeding support to social setting/peers |
| Motivation | |  |  |
| Plans | Educate, Train to form clearer personal rules/action plans, and train to remember and apply rules when needed | Have clear plans about how will breastfeed and manage problems | Have clear plans about how will support breastfeeding and manage issues |
| Evaluations | Educate or Persuade to create more positive beliefs about desired, and negative ones about undesired behaviour | Have to want to breastfeed and get a sense of satisfaction from doing it  Have to appreciate the negative consequences of formula feeding and see why not an equal alternative | Have to want to breastfeed and get a sense of satisfaction from doing it  Have to appreciate the negative consequences of formula feeding and see why not an equal alternative |
| Motives | Persuade, Incentivise, Coerce, Model or Enable to feel positively about the desired behaviour or weaken the undesired one | Have a strong sense that they should breastfeed | Have strong sense that their partner should breastfeed |
| Impulses/Inhibition | Train or Enable to strengthen habitual engagement in desired behaviour or weaken the undesired one | Get into a pattern of breastfeeding without having to think about it | Get into a pattern of supporting partner to breastfeed without having to think about it |
| Responses | Model desired behaviour to induce automatic imitation | Surround themselves with positive breastfeeding models | Surround themselves with positive breastfeeding supporter models |

**Behavioural specification pathway**

|  | **Who** | **What** | **Resources** | **Where** | **When** | **Measured by** |
| --- | --- | --- | --- | --- | --- | --- |
| Know why breastfeeding is important and how to do it | Lactation consultant/midwife | Antenatal class | Videos and slides;  Information sheets | In hospital | 28-36 weeks | Iowa Infant feeding attitudes scale – completed at baseline, late pregnancy and 6 weeks, 3 months, 6 months postpartum;  BAPT Survey |
| Learn how to breastfeeding and negotiate everyday problems such as maintaining social support | Lactation consultant/midwife | Postpartum (PP) 1:1 session;  PP Drop-in clinic;  PP lactation consultant phone calls hotline | N/A | In hospital; Outpatients department (OPD) | Immediately  postpartum;  Weekly until 6 weeks | Lactation consultant checklists;  BAPT Survey;  Latch assessment included in lactation consultant checklist;  Hughes Breastfeeding Support Scale |
| Build self-efficacy and physical health for breastfeeding | Lactation consultant/midwife | AN class;  Postpartum (PP) 1:1 session;  PP drop-in clinic;  PP lactation consultant phone calls hotline | Videos and slides;  Information sheets;  Problem solving; conversations | In hospital; Outpatients department (OPD) | 28-36 weeks;  Immediately  postpartum;  Weekly until 6 weeks | Breastfeeding Self-Efficacy Scale-Short Form (BSES-SF) |
| Build self-efficacy for longer term breastfeeding over time in the face of a formula feeding-culture | Lactation consultant/midwife | Postpartum (PP) 1:1 session;  PP Drop-in clinic;  PP lactation consultant phone calls hotline | N/A | Outpatients department (OPD) | Immediately  postpartum;  Weekly until 6 weeks | Breastfeeding Self-Efficacy Scale-Short Form (BSES-SF) |
|  |  |  |  |  |  |  |
| Create dedicated time for breastfeeding (partner support essential) | Lactation consultant/midwife | Antenatal clinic | Videos and slides;  Information sheets | In hospital | 28-36 weeks | Partner questionnaire |
| Acquire suitable breastfeeding supports if needed e.g. pillow, pump, creams, shields, clothing | Lactation consultant/midwife | Antenatal clinic | Videos and slides;  Information sheets | In hospital | 28-36 weeks | Partner questionnaire |
| Identify ways to make breastfeeding easier so can access facilities when needed e.g. at work, out socially or reminders at strategic times | Lactation consultant/midwife | Antenatal clinic | Videos and slides;  Information sheets | In hospital | 28-36 weeks | Partner questionnaire |
| Have supporters around them | Lactation consultant/midwife | Antenatal clinic | Videos and slides;  Information sheets | In hospital | 28-36 weeks | Partner questionnaire |
|  |  |  |  |  |  |  |
| Have clear plans about how will breastfeeding and manage problems | Lactation consultant/midwife | Antenatal class;  Postpartum (PP) 1:1 session;  PP drop-in clinic;  PP lactation consultant phone calls hotline | Videos and slides;  Information sheets;  Problem solving conversations | In hospital; Outpatients department (OPD) | 28-36 weeks;  Immediately postpartum;  Weekly until 6 weeks | Breastfeeding Self-Efficacy Scale-Short Form (BSES-SF) |
| Have to want to breastfeeding and get a sense of satisfaction from doing it  Have to appreciate the negative consequences of formula feeding and see why not an equal alternative | Lactation consultant/midwife | Antenatal class | Videos and slides;  Information sheets | In hospital | 28-36 weeks | Iowa Infant feeding attitudes scale |
| Have a strong sense that they should breastfeed | Lactation consultant/midwife | Antenatal clinic | Videos and slides;  Information sheets | In hospital | 28-36 weeks | Iowa Infant feeding attitudes scale |
| Get into a pattern of breastfeeding without having to think about it | Lactation consultant/midwife | Postpartum (PP) 1:1 session;  PP drop-in clinic;  PP lactation consultant phone calls |  | In hospital; Outpatients department (OPD) | Postpartum | Breastfeeding Self-Efficacy Scale-Short Form (BSES-SF) |
| Surround themselves with positive breastfeeding models | Lactation consultant/midwife | Antenatal Class | Videos and slides  Information sheets | In hospital | 28-36 weeks | Breastfeeding Attrition Prediction Tool (BAPT) |

**Appendix C**

**Antenatal Class Checklist Top of Form**

General

| Breastfeeding is normal way to feed baby |  |
| --- | --- |
| What partners can do (stars) |  |
| Recommendations |  |
| Importance and Benefits |  |
| Skin to skin contact |  |
|  |  |

Basics of Breastfeeding, the practical aspects

| First Few Days | |
| --- | --- |
| When milk 'comes in' |  |
| Normal baby weight loss |  |

| Recognizing hunger cues |  |
| --- | --- |
| Latching the baby |  |
|  |  |

| Good Positions | |
| --- | --- |
| Cross Cradle Position |  |
| Cradle Hold |  |
| Rugby Hold |  |
| Side Lying Position |  |
| Laid Back Breastfeeding |  |
|  |  |

| Knowing Baby is Getting Enough | |
| --- | --- |
| Newborn Stomach Capacity |  |
| Newborn Urine Output |  |
| Newborn stools |  |

| Additional tips |  |
| --- | --- |

| Hand expression |  |
| --- | --- |
| Be Prepared |  |
| Why babies cry |  |
| Winding your baby |  |

Breastfeeding Support

| What partners and others can do |  |
| --- | --- |
| What partners and others should avoid |  |

Challenges

| The Second Night |  |
| --- | --- |
| Positive bodies - mothers make milk |  |
| Nipple issues |  |
| Small mouth and large areola |  |
| Latch Aids |  |
| Engorged breasts |  |
| Mastitis |  |
| Feeding in public and/or with family or friends |  |
| Tongue-tie |  |
|  |  |

Other

| Any other notes? |
| --- |

Bottom of Form

**Appendix D**

**Postnatal Assessment Checklist**

Record Details

| Date * | (dd/mm/yyyy) |
| --- | --- |

**Location**

| Where was this assessment carried out? |
| --- |

| Bedside |  |
| --- | --- |
| Postnatal Clinic |  |
| Phone Call |  |
| Other |  |

| If other, please specify |
| --- |
|  |

**Education Checklist**

| Breast assessment |  |
| --- | --- |
| Infant oral assessment |  |
| What to expect in the early days |  |
| Knowing, understanding feeding cues and responding accordingly |  |
| How to position attach and recognize baby is effectively feeding |  |
| Ensuring a minimum of 8 feeds/24 hours and offering both breasts each feed |  |
| Knowing how to hand express |  |
| Follow up support for Latch On study and contact details for support groups |  |

| Any other comment? |
| --- |
|  |

**Newborn Feeding**

| Feeding method of newborn |  |
| --- | --- |
| Feed Type |  |
| Oral Intake | (ml) |
| Formula Type |  |
| Feeding Tolerance |  |
| Maternal Breastfeeding Assistance? |  |
| Feeding Difficulty Factors discussed? |  |
| Amount of Time for Feeding |  |
| Bottle DEMO |  |

**Breastfeeding Assessment**

| Length of time Left |  |
| --- | --- |
| Length of time Right |  |
| Nipple post feeding, Left |  |
| Nipple post feeding, Right |  |
| Hold Type |  |
| Hold assistance |  |
| Newborn behaviour during feeding |  |
| Breastfeeding count per 24 hours |  |

**Issues or Challenges Assessed**

| Baby feeds too often |  |
| --- | --- |
| Baby not breastfeeding well |  |
| Baby sleepy |  |
| Breast augmentation |  |
| Breast reduction |  |
| Difficulty in establishing breastfeeding |  |
| Flat nipples |  |
| Hyperbilirubinemia |  |
| Infant weight loss 7-10% |  |
| Inverted nipples |  |
| Maternal or infant medical condition |  |
| Maternal questions |  |
| Baby in the NICU or special care unit |  |
| No breast milk |  |
| Pain with latch |  |
| Pump and bottle feed breast milk |  |
| Pumping |  |
| Sore nipples |  |
| Using breast milk and formula |  |
| Using nipple shield |  |
| Using pacifier |  |

**Latch Assessment**

| Assessment | | 0 | 1 | 2 | Score |
| --- | --- | --- | --- | --- | --- |
| L | Latch On | Too sleepy or reluctant No sustained latch or suck achieved  | Repeated attempts for sustained latch or suck Hold nipple in mouth Stimulate to suck  | Grasps breast Toungue down Lips flanged Rhythmical Sucking  |  |
| A | Audible swallowing | None  | A few with stimulation  | Spontaneous and intermittent/frequent  |  |
| T | Type of nipple | Inverted  | Flat  | Averted  |  |
| C | Comfort | Engorged Cracked, bleeding, large blisters or bruises Severe discomfort  | Filling Reddened, small blisters or bruises  | Soft, not tender  |  |
| H | Hold Positioning | Full assist  | Minimal assist  | No assist  |  |
| Total LATCH score: | | | | |  |

| Comment on LATCH score |
| --- |

**Appendix E**

**Drop-in Clinic Checklist**

**Record Details**

| Date * | (dd/mm/yyyy) |
| --- | --- |

**Baby Details**

| Baby Age: | weeks | days |
| --- | --- | --- |

| Baby Birth Weight: | kg | OR | lbs | oz |
| --- | --- | --- | --- | --- |
| Baby Current Weight: | kg | OR | lbs | oz |

**Medical and Breastfeeding History**

| Relevant Medical and Obstetric History |
| --- |
| Breastfeeding History |

**Breastfeeding Challenges Discussed**

| Baby feeds too often |  |
| --- | --- |
| Baby not breastfeeding well |  |
| Baby sleepy |  |
| Breast augmentation |  |
| Breast reduction |  |
| Difficulty in establishing breastfeeding |  |
| Flat nipples |  |
| Hyperbilirubinemia |  |
| Infant weight loss 7-10% |  |
| Inverted nipples |  |
| Maternal or infant medical condition |  |
| Maternal questions |  |
| Baby in the NICU or special care unit |  |
| No breast milk |  |
| Pain with latch |  |
| Pump and bottle feed breast milk |  |
| Pumping |  |
| Sore nipples |  |
| Using breast milk and formula |  |
| Using nipple shield |  |
| Using pacifier |  |
| Maternal sleep |  |
| Support at home |  |
| Tongue tie |  |

**Self-Efficacy**

| How is she dealing with breastfeeding among friends and family? |  |
| --- | --- |
| How is she dealing with breastfeeding out in public? |  |
| Does she feel supported? |  |
| Is she managing to negotiate tricky situations (e.g. family saying negative things, cafe, public situations)? |  |
| How is she finding managing the time that breastfeeding is taking in the day? |  |
| Is her partner helping out at home? |  |
| Is she starting to think about how she will manage when going back to work? |  |
| Notes | |

**Latch Assessment**

| Assessment | | 0 | 1 | 2 | Score |
| --- | --- | --- | --- | --- | --- |
| L | Latch On | Too sleepy or reluctant No sustained latch or suck achieved | Repeated attempts for sustained latch or suck Hold nipple in mouth Stimulate to suck | Grasps breast Toungue down Lips flanged Rhythmical Sucking |  |
| A | Audible swallowing | None | A few with stimulation | Spontaneous and intermittent/frequent |  |
| T | Type of nipple | Inverted | Flat | Averted |  |
| C | Comfort | Engorged Cracked, bleeding, large blisters or bruises Severe discomfort | Filling Reddened, small blisters or bruises | Soft, not tender |  |
| H | Hold Positioning | Full assist | Minimal assist | No assist |  |
| **Total LATCH score:** | | | | |  |

| **Comment on LATCH score** |
| --- |

**Other**

| Swabs/Samples taken |  |
| --- | --- |
| If yes, details | |
|  | |

| Expressing |  |
| --- | --- |
| Supplementing |  |
|  |  |

| Plan (brief)   \| Follow-up call discussed? \|  \| \| --- \| --- \| \| Follow-up at drop in clinic next week discussed? \|  \| |
| --- | --- | --- | --- | --- |

**Appendix F**

**Follow-up Phone Call Checklist**

**Record Details**

| Date * | (dd/mm/yyyy) |
| --- | --- |

**Medical and Breastfeeding History**

| Breastfeeding history |
| --- |

**Breastfeeding Challenges Discussed**

| Baby feeds too often |  |
| --- | --- |
| Baby not breastfeeding well |  |
| Baby sleepy |  |
| Breast augmentation |  |
| Breast reduction |  |
| Difficulty in establishing breastfeeding |  |
| Flat nipples |  |
| Hyperbilirubinemia |  |
| Infant weight loss 7-10% |  |
| Inverted nipples |  |
| Maternal or infant medical condition |  |
| Maternal questions |  |
| Baby in the NICU or special care unit |  |
| No breast milk |  |
| Pain with latch |  |
| Pump and bottle feed breast milk |  |
| Pumping |  |
| Sore nipples |  |
| Using breast milk and formula |  |
| Using nipple shield |  |
| Using pacifier |  |
| Maternal sleep |  |
| Support at home |  |
| Tongue tie |  |

**Self-Efficacy**

| How is she dealing with breastfeeding among friends and family? |  |
| --- | --- |
| How is she dealing with breastfeeding out in public? |  |
| Does she feel supported? |  |
| Is she managing to negotiate tricky situations (e.g. family saying negative things, cafe, public situations)? |  |
| How is she finding managing the time that breastfeeding is taking in the day? |  |
| Is her partner helping out at home? |  |
| Is she starting to think about how she will manage when going back to work? |  |
| Notes | |

**Other**

| Expressing |  |
| --- | --- |
| Supplementing |  |

| Plan (brief) |
| --- |

| Follow-up call discussed? |  |
| --- | --- |
| Follow-up at drop in clinic next week discussed? |  |
